# Lipidomic and sterolomic profiles of different brain regions in the mouse model of Alzheimer’s disease

**DOI:** 10.4103/NRR.NRR-D-24-00975

**Published:** 2025-02-24

**Authors:** Xingsen Zhao, Liqun Chen, Liangjian Ma, Xiaohui Liu, Zhongkai Cao, Xiangjun Chen, Lidan Hu

**Affiliations:** 1 Department of Nephrology, The Children’s Hospital, Zhejiang University School of Medicine, National Clinical Research Center for Child Health, Hangzhou, Zhejiang Province, China; 2 Institute of Biotechnology, Xianghu Laboratory, Hangzhou, Zhejiang Province, China; 3 Academy of Medical Engineering and Translational Medicine, Medical College, Tianjin University, Tianjin, China; 4 The First Clinical Medical College of Lanzhou University, Lanzhou, Gansu Province, China; 5 Metabolomics and Lipidomics Center of Tsinghua University, Beijing, China; 6 Institute of Translational Medicine, Zhejiang University School of Medicine, Hangzhou, Zhejiang Province, China

**Keywords:** Alzheimer’s disease, Alzheimer’s disease mouse model, brain lipids, dyslipidemias, lipidomic, metabolism, neurodegenerative disease, sterol

## Abstract

Alzheimer’s disease is the most common cause of dementia. Although increasing evidence suggests that disruptions in lipid metabolism are closely associated with the disease, the overall profile of lipid and sterol changes that occur in the brain during Alzheimer’s disease remains unclear. In this study, we compared brain tissues extracted from 32-week-old male wild-type mice and 5×FAD transgenic Alzheimer’s disease model mice, which carry mutations in the amyloid precursor protein (*APP*) and presenilin 1 (*PS1*) genes. Using untargeted lipidomics and sterolomics techniques, we investigated the metabolic profiles of lipids, with a focus on sterols specifically, in three brain regions: cerebellum, hippocampus, and olfactory bulb. Our results revealed significant alterations in various lipids, particularly in the hippocampus and olfactory bulb, suggesting changes in energy levels in these regions. Further pathway analysis indicated notable disruptions in key metabolic processes, particularly those related to fatty acids and cell membrane components. Additionally, we observed decreased expression of 15 genes involved in lipid and sterol regulation. Collectively, these findings provide new insights into how imbalances in lipid and sterol metabolism may contribute to the progression of Alzheimer’s disease, highlighting potential metabolic pathways involved in the development of this debilitating disease.

## Graphical abstract

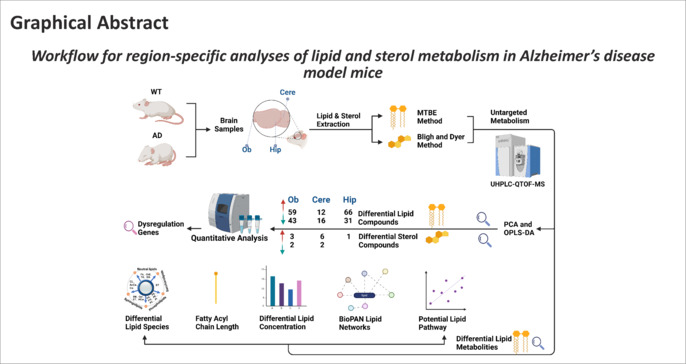

## Introduction

Alzheimer’s disease (AD), a progressive neurodegenerative disease of the central nervous system and the most common type of dementia of old age, is characterized by irreversible cognitive and behavioral dysfunction (Bondi et al., 2017; Lane et al., 2018; Soria Lopez et al., 2019). Neurodegeneration in AD is initiated by the deposition of amyloid-β (Aβ) plaques derived from amyloid precursor protein (APP), which lead to neuroinflammation and neuronal death (Long and Holtzman, 2019). The International Alzheimer’s Disease Association reported that, as of 2020, the number of AD cases in the United States was expected to increase to approximately 14 million in 2060, with a current annual treatment cost of $1 trillion expected to double by 2030 (Skaria, 2022). The rising prevalence of AD presents a substantial challenge to economic and social development. The advancement of multiomics technologies has enabled the use of next-generation sequencing, single-cell transcriptomic analysis, and comprehensive studies of transcriptomes, proteomes, metabolomes, and lipidomes to elucidate the pathophysiologic characteristics of AD (Mathys et al., 2019; Fitzner et al., 2020; Morabito et al., 2020). Various factors, including age, sex, education level, genetic variations, and metabolic conditions, such as diabetes, hypertension, and obesity, have all been associated with an increased risk of developing AD (Qiu et al., 2009; Zhao and Li, 2020). However, the complex and multidimensional nature of AD etiology has hindered the discovery of the precise relationships between these factors.

Lipids, which include a subclass of sterols, play critical roles in maintaining the structure and function of cell membranes and regulate various cellular processes, including signaling, metabolism, and inflammation (Santos and Preta, 2018; Leuti et al., 2020; Zhang et al., 2025). The brain is one of the most lipid-abundant organs, with lipids constituting approximately 60% of its dry weight (Hamilton et al., 2007). The robust body of literature encompassing genetic and lipidomic investigations has consistently pointed towards impaired lipid metabolism in individuals with AD (El Gaamouch et al., 2016; Rebeck, 2017; Yin, 2023). Nevertheless, a significant knowledge gap remains concerning the comprehensive lipid profile and underlying lipidomic pathways of the disease. Cholesterol, also enriched in the brain, is essential for normal brain function and development, playing a pivotal role in the formation of the axon myelin sheath, which facilitates optimal neuroconductivity (Korade et al., 2022). Cholesterol is also crucial for axon regeneration following nerve injury (Zhang and Liu, 2015; Berghoff et al., 2022; Huang et al., 2025). Furthermore, disrupted cholesterol metabolism induces neuronal cell toxicity and impedes key processes, such as axonogenesis, synaptogenesis, and action potential generation, thereby greatly contributing to diverse neurological and neuropsychiatric disorders (Cheon, 2023; Tian et al., 2025). Emerging evidence suggests that a dietary pattern characterized by diminished consumption of high-fat foods and sweets may help to mitigate the occurrence of AD (Ruan et al., 2018). The excess free cholesterol in cells can be converted into cholesterol esters by the action of sterol oxygen-acyltransferase 1 enzyme (ACAT1). An elevated cholesterol ester level increases the release of Aβ protein in cultured cells *in vitro*, while pharmacological inhibition of ACAT1 lowers the levels of Aβ protein and cholesterol ester. Knockout of *Acat1* reduced Aβ pathology and cognitive impairment in AD mouse models (Puglielli et al., 2001; Hutter-Paier et al., 2004; Bryleva et al., 2010). The use of cholesterol-extracting compounds such as β-methylcyclodextrin can reduce membrane levels of cholesterol, inhibit the activity of β-secretase 1 and γ-secretase, and increase the production of Aβ in cells (Hartmann et al., 2007; Vetrivel and Thinakaran, 2010). Sphingomyelinase is an enzyme that mediates the conversion of sphingomyelin (SM) to ceramide. Inhibition of intracellular sphingomyelinase activity leads to SM accumulation, which can inhibit γ-secretase activity and reduce the production of Aβ protein (Grimm et al., 2005). A previous study has also shown that the phospholipase D pathway can regulate the production of Aβ (Oliveira and Di Paolo, 2010). Despite the detection of abnormal lipid metabolism in AD in early research, and our current understanding of its significant impact on the pathological process of AD, specific lipid analyses of different regions of the brain in AD are lacking. Analysis of the lipid changes in AD identified by lipidomics could be useful to predict the progression of the disease. Lipidomic studies reveal relationships between biochemical pathways and provide mechanistic insights necessary to determine the molecular basis of cellular or organismal changes. We hypothesized that, within lipid metabolism, significant alterations in sterol metabolism specifically may be involved in AD. However, past efforts to elucidate brain sterol metabolism have been limited.

Here, we sought to identify specific sterol and other lipid species that significantly associate with AD occurrence by measuring changes in lipid levels in three areas of the brain in AD mice: cerebellum (Cere), hippocampus (Hip), and olfactory bulb (Ob). Subsequently, we employed pathway analysis to help elucidate the underlying mechanisms contributing to these alterations. To deepen our understanding of the involved pathways, we used quantitative PCR (qPCR) to quantify the expression levels of various genes associated with these mechanisms. By offering comprehensive insights into the intricate dynamics of lipid and sterol metabolism in the context of AD, our findings will contribute to the establishment of a solid theoretical foundation for the development of drugs and nutritional health management approaches.

## Methods

### Animals

All experiments were conducted in accordance with the protocols approved by the Ethical Review Committee of Laboratory Animal Welfare, Zhejiang University on April 27, 2023 (Approval No. ZJU20230105), and were designed and reported in accordance with the Animal Research: Reporting of *In Vivo* Experiments (ARRIVE) guidelines (Percie du Sert et al., 2020). All mice were bred and maintained in the animal facility of Zhejiang University, on a 12-hour light/dark cycle, and were fed a standard diet. We used male double-transgenic mice that co-express mutated human APP and presenilin 1 (PS1) genes (homozygous 5×FAD; *APP* K670N/M671L-I716V-V717I + *PS1* M146L-L286V, Jackson Laboratory Strain #034840, RRID: MMRRC_034840-JAX), which are widely applied to model familial AD. Male heterozygous 5×FAD littermates aged 32 weeks, and adult male wild-type (WT) C57BL/6J mice of same ages, were used as controls (*n* = 8–13). DNA from each mouse was extracted and sequenced. For euthanization, mice were anesthetized with 2%–5% isoflurane (RWD, Shenzhen, China). Ob, Hip, and Cere tissues were isolated from the mouse brain on ice, and frozen in liquid nitrogen for subsequent storage at –80°C (**Additional Figure 1**).

### Immunofluorescence staining

Brain tissues of mice anesthetized with isoflurane were perfused with 4% paraformaldehyde, fixed in 4% polyformaldehyde at 4°C for 24 hours, then transferred to a 30% sucrose solution at 4°C for dehydration. The tissues were sectioned at a thickness of 30 μm using a frozen slicer. After washing with 1× phosphate-buffered saline (PBS), the brain slices were blocked with PBS containing 3% normal goat serum and 0.1% Triton X-100 at room temperature (25°C) for 1 hour. The sections were incubated with primary antibodies against Aβ (1:2000 dilution; CST, Boston, MA, USA, Cat# 2454s, RRID: AB_2056585) overnight at 4°C. On the second day, the sections were incubated with 4’,6-diamidino-2-phenylindole (DAPI) (1:10,000; Sigma, St. Louis, MO, USA, Cat# D8417) and Alexa Fluor 488-conjugated goat anti-rabbit secondary antibody (1:3000, Thermo Fisher Scientific, MA, USA, Cat# A11008, RRID: AB_143165) at room temperature for 1 hour. Images were taken with an Olympus FV3000 confocal microscope (Tokyo, Japan).

### Lipid extraction

Lipids were extracted using the methyl-tert-butyl ether (MTBE) method, as described previously (Matyash et al., 2008). Briefly, 17 mg each of Cere, Hip, and Ob tissues from one mouse were lysed in 1 mL PBS buffer, to which methanol was added proportionally (1.5 mL methanol per 200 μL sample aliquot) and vortexed. Next, 5 mL MTBE was added to each sample, and the mixtures were incubated for 1 hour at room temperature in a shaker. Phase separation was induced by adding 1.25 mL mass spectrometry (MS)-grade water. Upon 10 minutes of incubation at room temperature, the samples were centrifuged at 1000 × *g* for 10 minutes. The upper (organic) phases were collected, and the lower phases were re-extracted with 2 mL of a solvent mixture comprising the expected composition of the upper phase (obtained by mixing MTBE/methanol/water at 10:3:2.5 (v/v/v) and collecting the upper phase). Combined organic phases were dried in a vacuum centrifuge (Thermo Fisher Scientific, Cat# SPD140P2).

### Sterol extraction

Sterol extraction was performed using the Bligh and Dyer method (Bligh and Dyer, 1959). Briefly, tissues were lysed three times by ultrasound in cold PBS, followed by four cycles of freezing and thawing in liquid nitrogen. The lysed tissues were added to an extraction solution (methanol: chloroform = 2:1) at a volume ratio between the aqueous phase and the organic phase of 1:4. The samples were vortex-agitated for 1 minute and incubated for 2 minutes at 4°C a total of three times. The organic and aqueous phases were separated by centrifugation at 1000 × *g* for 15 minutes. After separation, the lower organic phases containing sterols were collected. All operations were performed using glass tubes or glass syringes to avoid polymer contamination. The collected organic phases were dried at 50°C under nitrogen flow. The dried samples were stored at –80°C for further liquid chromatography (LC)-MS analysis.

### Lipidomics data acquisition and analysis

Lipids were re-dissolved in 100 μL methanol/chloroform (v:v = 1:1) containing 2 µg/mL each of the following internal standards: lysophosphatidylcholine (14:0), lysophosphatidylserine (14:0), phosphatidylcholine (14:0/14:0), phosphatidylethanolamine (PE) (14:0/14:0), phosphatidylserine (14:0/14:0), and fatty acid (FA) (18:0)-D35. A reversed-phase C18 column (2.1 × 100 mm; Waters Corporation, Milford, MA, USA) was used for MS separation, following a previous study (Hu et al., 2024) with modification. Lipid analysis was performed using a Q Exactive Orbitrap (Thermo Fisher Scientific). Data-dependent acquisition was applied with resolutions of 70000 and 17500 for MS and tandem MS (MS/MS) scans, respectively. The top 10 most intense precursors were selected for fragmentation, with a 7-second duration of dynamic exclusion. Detailed parameters included the following: spray voltage, 3.2 kV for positive and 2.8 kV for negative; capillary temperature, 320°C; auxiliary gas flow rate, 10; mass range (m/z), 240–2000 for positive and 200–2000 for negative. Mobile phase A was prepared by dissolving 0.77 g ammonium acetate in 400 mL high-performance liquid chromatography (HPLC)-grade water, followed by the addition of 600 mL HPLC-grade acetonitrile. Mobile phase B was prepared by mixing 100 mL acetonitrile with 900 mL isopropanol. The gradient was as follows: 0 minutes, 37% B; 1.5 minutes, 37% B; 4 minutes, 45% B; 5 minutes, 52% B; 8 minutes, 58% B; 11 minutes, 66% B; 14 minutes, 70% B; 18 minutes, 75% B; 20 minutes, 98% B; 22 minutes, 98% B; 22.1 minutes, 37% B; and 25 minutes, 37% B. Lipids were identified and quantified using LipidSearch 4.1.30 (Thermo Fisher Scientific), and lipid assignment was confirmed manually based on MS/MS fragmentation. Mass tolerances of 5 ppm and 10 ppm were applied for precursor and product ions, respectively. Only lipids with chromatographic peak areas > 5 × 10^6^ were considered to be confidently identified. A retention time shift of 0.15 minutes was allowed for peak alignment.

### Sterol data acquisition

The ultra-HPLC (UPLC) system was coupled to a Q-Exactive HFX Orbitrap mass spectrometer (Thermo Fisher Scientific) equipped with an atmospheric pressure chemical ionization probe. A 1 µL volume of supernatant was loaded onto a Kinetex® Biphenyl column (2.1 × 150 mm, 2.6 µm, Phenomenex, Torrance, CA, USA) for the positive mode. The sample was then eluted to the mass spectrometer with acetonitrile containing 0.1% formic acid as the eluent, from 80% to 99% within 9.5 minutes. The stationary phase was aqueous with 0.1% formic acid. Data were acquired in positive ion mode with a mass range of 80–1200 m/z, using data-dependent MS/MS acquisition. The full scan and fragment spectra were collected with resolutions of 60,000 and 15,000, respectively. The source parameters were as follows: spray current, 8 μA; capillary temperature, 320°C; heater temperature, 300°C; sheath gas flow rate, 35 Arb; auxiliary gas flow rate, 10 Arb. Data analysis was performed by Xcalibur 4.4 software (Thermo Fisher Scientific). Potential lipid biomarkers were identified by matching the experimental retention time (±0.1 minute), accurate mass, and tandem mass spectra with those available in the Human Metabolome Database (HMDB) (https://hmdb.ca/spectra/ms/search) (Wishart et al., 2007, 2009, 2022; Hu et al., 2024), using a mass accuracy of ±3 ppm for fragment ions, excluding the lipids. The results were confirmed with commercial standard compounds when available.

### Quantitative polymerase chain reaction

Total RNA was extracted from brain tissue using TRIzol reagent (Thermo Fisher Scientific, Cat# 15596018) and purified with chloroform, in accordance with the manufacturer’s protocol. The concentration of RNA was quantified using a NanoDrop spectrophotometer 1000 (Thermo Fisher Scientific). The total RNA (0.5 µg) was reverse transcribed, and a qPCR assay was performed using SYBR Green (Vazyme, Nanjing, China, Cat# Q412-02). The primers for the targeted genes are shown in **Additional Table 1**. The relative abundance of mRNA was determined by normalization to 18S rRNA levels.

**Additional Table 1:** The primer sequences for quantitative polymerase chain reaction

Gene	Primer sequence (5’-3’)
*Apoc*	F: TCCTGTCCTGATTGTGGTCGT R: CCAAAGTGTTCCCAAACTCCTT
*Ch25h*	F: TGCTACAACGGTTCGGAGC R: AGAAGCCCACGTAAGTGATGAT
*Ldir*	F: TGACTCAGACGAACAAGGCTG R: ATCTAGGCAATCTCGGTCTCC
*Sorl1*	F: AGCAGGAGGGAGTCGAGAC R: GTTCCTAGCCGGAGATCGC
*LSS*	F: TCGTGGGGGACCCTATAAAAC R: CGTCCTCCGCTTGATAATAAGTC
*Idi1*	F: ACCAGCCATCTTGATGAAAAACA R: CAGCAACTATTGGTGAAACAACC
*Abca1*	F: GCTTGTTGGCCTCAGTTAAGG R: GTAGCTCAGGCGTACAGAGAT
*Sqle*	F: ATAAGAAATGCGGGGATGTCAC R: ATATCCGAGAAGGCAGCGAAC
*Fdft1*	F: ATGGAGTTCGTCAAGTGTCTAGG R: CGTGCCGTATGTCCCCATC
*Nsdh1*	F: TCATGGTGAATCAAAGCGAGG R: CCGGGGGTTATCAAAGCCTTG
*Hmgcs1*	F: AACTGGTGCAGAAATCTCTAGC R: GGTTGAATAGCTCAGAACTAGCC
*Fdps*	F: GGAGGTCCTAGAGTACAATGCC R: AAGCCTGGAGCAGTTCTACAC
*Mvd*	F: ATGGCCTCAGAAAAGCCTCAG R: TGGTCGTTTTTAGCTGGTCCT
*Soat1*	F: GAAACCGGCTGTCAAAATCTGG R: TGTGACCATTTCTGTATGTGTCC
*Dhcr24*	F: CTCTGGGTGCGAGTGAAGG R: TTCCCGGACCTGTTTCTGGAT
*APP*	F: GACCCATCCCCACTTTGTGA R: CAGGAACGAGAAGGGCATCA
*Psen1*	F: GGTCATCCATGCCTGGCTTATTA R: GCAACAGTAATGTAGTCCACAGC
*18s*	F: CGGCTACCACATCCAAGGAA R: CCTGTATTGTTATTTTTCGTCACTACCT

F: Forward; R: reverse.

### Statistical analysis

Peak detection, alignment, and integration of the raw data were carried out using Profinder 10.0 software (Agilent Technologies, Santa Clara, CA, USA). The lipid data identified by LC-MS were analyzed using MetaboAnalyst (http://www.metaboanalyst.ca/), employing the LIPID MAPS database (https://www.lipidmaps.org/biopan/) (Conroy et al., 2024). Principal component analysis (PCA) and orthogonal partial least-squares discriminant analysis (OPLS-DA) were performed using MetaboAnalyst. Data were scaled to unit variance prior to conducting the PCA. Metabolite enrichment data were log-transformed before being subjected to pathway analysis. A fold change (FC) ≥ 2 or ≤ 0.5 and a false discovery rate < 0.05 were used as cutoff values to select significant target lipids. Statistical comparisons among the different groups were made using unpaired Student’s *t*-tests and one-way analysis of variance in Graph Pad Prism (Version 8.0, GraphPad Software, Boston, MA, USA, www.graphpad.com). The samples were categorized by FA chain length into medium- and long-chain FAs (C12 to C24) and long-chain FAs (> C24), and the saturation level of peptide bonds (C=O) was determined. To further investigate the FA pathways in our sample cohort, we analyzed the data using the BioPAN tool available on the LIPID MAPS website (https://lipidmaps.org/tool/biopan/; Gaud et al., 2021). For sterol analysis, we screened potential compounds using the molecular weight (m/z) standards on the HMDB website to identify molecular differences (FC < 0.5 or > 2 [AD/WT] and *P* < 0.05) between the WT and AD groups in each brain region. After HMDB screening, we included the sterols and excluded the other lipids. *P* values < 0.05 were considered to indicate statistical signiﬁcance.

## Results

### Regional resolution and coverage of brain lipidomes in adult Alzheimer’s disease mice

**Figure 1:**
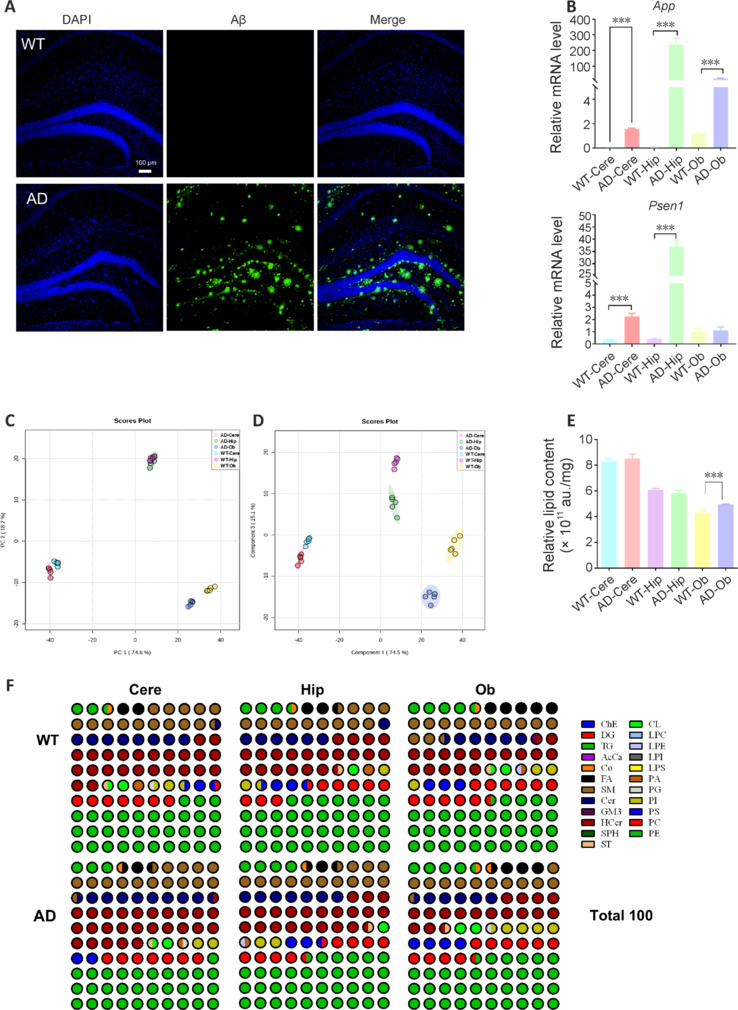
Construction of AD mice model and lipid-class distribution in mouse brain regions. (A) Immunofluorescence staining of brain sections. Aβ was higher expressed in AD group. Aβ: Green, stained by Alexa Fluor 488, DAPI: blue. Scale bar: 100 μm. (B) qPCR analysis of *App* and *Psen1* in AD and WT group, and is higher expressed in AD group. The relative abundance of mRNA was obtained by normalization to 18S levels. (C) Score plots of principal component analysis from Cere, Hip and Ob metabolite lipidomic spectra of WT and AD. (D) Score plots of orthogonal partial least-squares discriminant analysis from Cere, Hip, and Ob sample lipidomic spectra of WT and AD. (E) Total lipid (overall mean peak intensity of all lipid classes) between WT and AD. (F) Lipid species composition of WT and AD brain region tissue, displayed as parts of the whole. Detailed content of lipids is shown in Additional Table 2. For this study, male AD model groups and age-matched WT controls (*n* = 8–13) were used. Data are presented as mean ± SEM, unpaired Student’s *t*-test, ***P < 0.0001. AcCa: Acylcarnitine; AD: Alzheimer’s disease; Aβ: amyloid-β; Cere: cerebellum; Cer: ceramide; ChE: cholesteryl ester; CL: cardiolipin; Co: coenzyme; DAPI: 4′,6-diamidino-2-phenylindole; DG: diacylglycerol; FA: fatty acid; GM3: monosialotetrahexosylganglioside; HexCer: hexosylceramide; Hip: hippocampus; LPC: lysophosphatidylcholine; LPE: lysophosphatidylethanolamine; LPI: lysophosphatidylinositol; LPS: lysophosphatidylserine; Ob: olfactory bulb; PA: phosphatidic acid; PC: phosphatidylcholine; PE: phosphatidylethanolamine; PG: phosphatidylglycerol; PI: phosphatidylinositol; PS: phosphatidylserine; qPCR: quantitative polymerase chain reaction; SM: sphingomyelin; SPH: sphingosine; TG: triacylglycerol; WT: wild type.

For this study, we generated 5×FAD mice, an established transgenic model of AD. Immunofluorescence staining of brain sections and qPCR analysis for *App* and *Psen1* revealed the presence of Aβ deposits and high levels of *App* and *Psen1* expression in the brains of AD group mice (**Figure 1A** and **B**), indicating successful induction of the disease phenotype. To resolve the lipidomes of distinct regions of the adult mouse brain, we collected brain samples from specific anatomical structures, namely the Cere, Hip, and Ob from 32-week-old male mice. High-resolution MS determination of lipid profiles was carried out for all three brain regions of WT and AD mice. After lipid analysis and alignment with the LipidSearch dataset, we detected 2750 peaks in positive mode and 2030 peaks in negative mode. Data annotation revealed 589 lipid features in positive mode and 460 lipid features in negative mode. Following optimization of the analysis method and normalization of the data to internal standards, we ultimately selected 664 unique lipid species and 23 lipid classes for further analysis. The complete lipidomic dataset is presented in **Additional Table 2**. The data for each lipid were transformed into the percentage of moles per sample, allowing direct comparison of samples with different amounts of total lipids.

**Additional Table 2:** Lipid species percentage in distinct brain regions

Lipid species	WT-Cere	WT-Ob	WT-Hip	AD-Cere	AD-Ob	AD-Hip
ChE	0.003	0.064	0.005	0.003	0.044	0.015
DG	0.171	0.185	0.134	0.187	0.137	0.161
TG	2.538	4.261	3.21	2.726	4.492	3.92
AcCa	0.014	0.026	0.011	0.022	0.02	0.022
Co	0.494	0.712	0.537	0.495	0.648	0.519
FA	1.982	4.918	2.764	1.837	3.63	1.959
SM	14.22	12.333	12.509	15.359	11.32	13.435
Cer	8.525	5.705	6.906	8.993	5.631	7.146
GM3	0.01	0.079	0.025	0.011	0.07	0.036
HCer	24.144	16.715	20.324	25.45	16.553	21.424
SPH	0.007	0.021	0.01	0.005	0.016	0.011
ST	0.405	0.287	0.349	0.431	0.273	0.375
CL	1.47	1.842	1.316	1.258	1.926	1.074
LPC	0.01	0.019	0.014	0.009	0.015	0.021
LPE	0.076	0.35	0.082	0.025	0.035	0.147
LPI	0.002	0.004	0.002	0.002	0.01	0.002
LPS	0.003	0.016	0.005	0.003	0.007	0.005
PA	0.9	0.227	0.561	0.858	0.393	0.51
PG	0.323	0.331	0.216	0.279	0.358	0.193
PI	2.082	2.866	2.286	2.168	4.635	2.102
PS	1.883	2.839	3.023	1.866	3.571	2.62
PC	7.754	11.614	8.883	7.248	10.634	8.99
PE	32.984	34.587	36.829	30.766	35.583	35.31

AcCa: Acylcarnitine; AD: Alzheimer's disease; Cer: ceramide; Cere: cerebellum; ChE: cholesteryl ester; CL: cardiolipin; Co: coenzyme; DG: diacylglycerol; FA: fatty acid; GM3: monosialotetrahexosylganglioside; HCer: hexosylceramide; Hip: hippocampus; LPC: lysophosphatidylcholine; LPE: lysophosphatidylethanolamine; LPI: lysophosphatidylinositol; LPS: lysophosphatidylserine; Ob: olfactory bulb; PA: phosphatidic acid; PC: phosphatidylcholine; PE: phosphatidylethanolamine; PG: phosphatidylglycerol; PI: phosphatidylinositol; PS: phosphatidylserine; SM: sphingomyelin; SPH: sphingosine; ST: sterol lipid; TG: triacylglycerol; WT: wild type.

The biological replicates exhibited almost no variation, demonstrating excellent technical and biological reproducibility. The lipid profiles of the three brain regions were analyzed using PCA and OPLS-DA to explore the distinct lipid compositions in WT and AD mice. These analyses revealed clear clustering patterns indicative of significant differences in lipid composition between the Cere, Hip, and Ob in WT mice (**Figure 1C** and **D**), consistent with previous studies (Fitzner et al., 2020). AD mice showed a similar trend (**Figure 1C** and **D**). The PCA plots illustrated divergence in the lipid compositions of AD-affected brain regions between AD and WT mice, with the Ob region exhibiting the most marked differences (**Figure 1C**). In terms of absorbance units/mg of the same tissue mass, the total lipid content of the Cere was slightly higher than that of the Hip and Ob in both groups (Cere: **~**8.0 × 10^11^; Hip: **~**6.0 × 10^11^; Ob: **~**4.0 × 10^11^). However, the significantly higher total lipid content observed in the AD Ob compared with that in the WT Ob suggested that lipid metabolism may be particularly altered in this region compared with that in the Cere and Hip (**Figure 1E**). To make these findings more accessible, we included a summary diagram that outlines the key lipid changes across these regions in an intuitive manner (**Figure 1F**), illustrating the prevalences of various lipids in both the WT and AD datasets. Among these lipids, PE, SM, and hexosylceramide (HexCer) were the three most common classes in all groups of samples. However, distinct differences in lipid class profiles and patterns were observed among the three brain regions.

### Differences in lipid content of Alzheimer’s disease–affected regions of the brain

**Figure 2:**
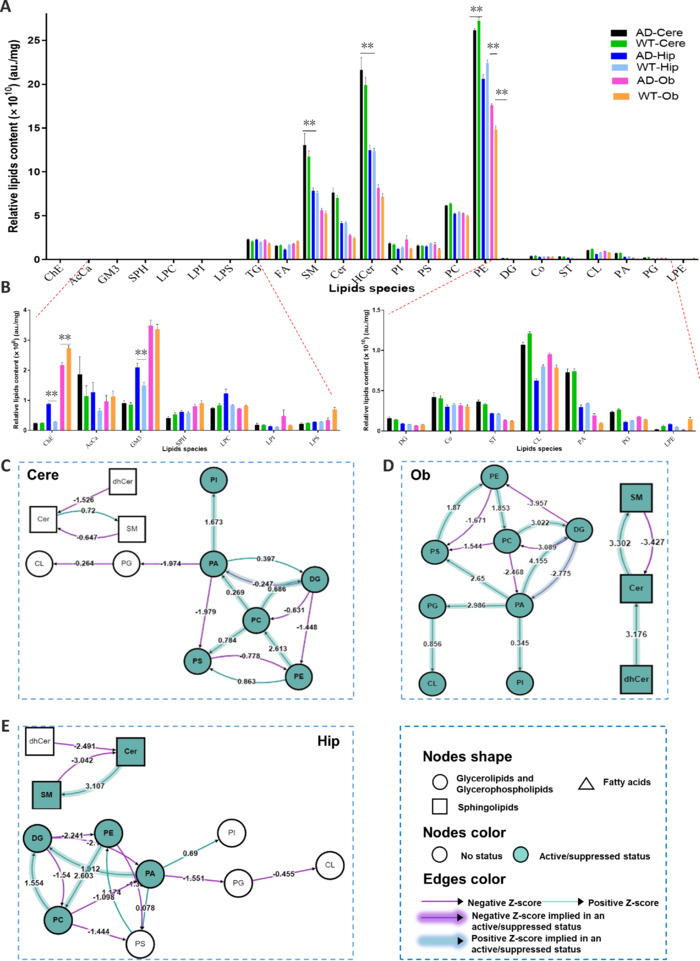
Differences in the concentrations of individual lipid classes in distinct brain regions. (A) Relative concentration analysis of the overall lipid species. For each lipid species, bars indicate the relative signal of each lipid species. (B) Relative concentration analysis of the low-content lipid species. **P < 0.01 (unpaired Student’s *t*-test). (C–E) Lipid network graphs exported from BioPAN for Cere (C), Ob (D) and Hip (E) compared to WT mice. Green nodes correspond to active lipids and green shaded arrows correspond to active pathways. Reactions with a positive Z score have green arrows while negative Z scores are color purple. Pathway options: AD condition of interest, WT control condition, lipid type, active status, subclass level, and reaction subset of lipid data, one-way analysis of variance, *P* value, and no paired-data. AD: Alzheimer’s disease; Cere: cerebellum; Hip: hippocampus; Ob: olfactory bulb; WT: wild type.

To better understand lipid function in AD, we compared the lipid profiles of different brain regions between AD and WT mice using lipidomics analysis. After normalizing the mean peak intensity to the tissue mass, we found that triglyceride phosphate (TG), FA, SM, ceramide, HexCer, phosphatidylinositol (PI), phosphatidylserine, phosphatidylcholine, and PE were the lipids with the highest content within all three brain regions in both AD and WT mice (**Figure 2A**). Notably, there were significant differences in the levels of specific lipid species between the two groups (**Additional Figure 1A–I**). Specifically, the levels of sphingolipids SM and HexaCer in the Cere were higher in the AD group than in the WT group. Additionally, in AD mice, the Hip region contained a lower level of PE and a higher level of cholesterol ester (ChE), while the Ob region had a higher level of PE and a lower level of ChE (**Figure 2B**), compared with WT mice. Interestingly, our results also revealed a difference in total lipids of the Cere and Ob regions between AD and WT mice (**Additional Figure 2A** and **G**). Next, we used the BioPAN tool to analyze the interconversion of lipids, which showed tissue-specific differences in sphingolipid metabolism between AD and WT mice (Gaud et al., 2021). Specifically, ceramide and SM were found to be inactive in the Cere region, but active in the Hip and Ob regions (SM, Z score = 3.107 in Hip and 3.302 in Ob) of AD mice (**Figure 2C–E**). This observation suggested a potential link between sphingolipid metabolism and AD progression in these brain regions. We also observed an accumulation of diacylglycerol (DG) in both the Hip and Ob regions that was not observed in the Cere region. Additionally, there was active phospholipid metabolism in the Ob region. Overall, our findings suggested that lipid metabolism is differentially altered in certain regions of the brain in AD mice, which may have implications for AD pathology.

### Regional differences in lipid species between Alzheimer’s disease and wild-type mice

**Figure 3:**
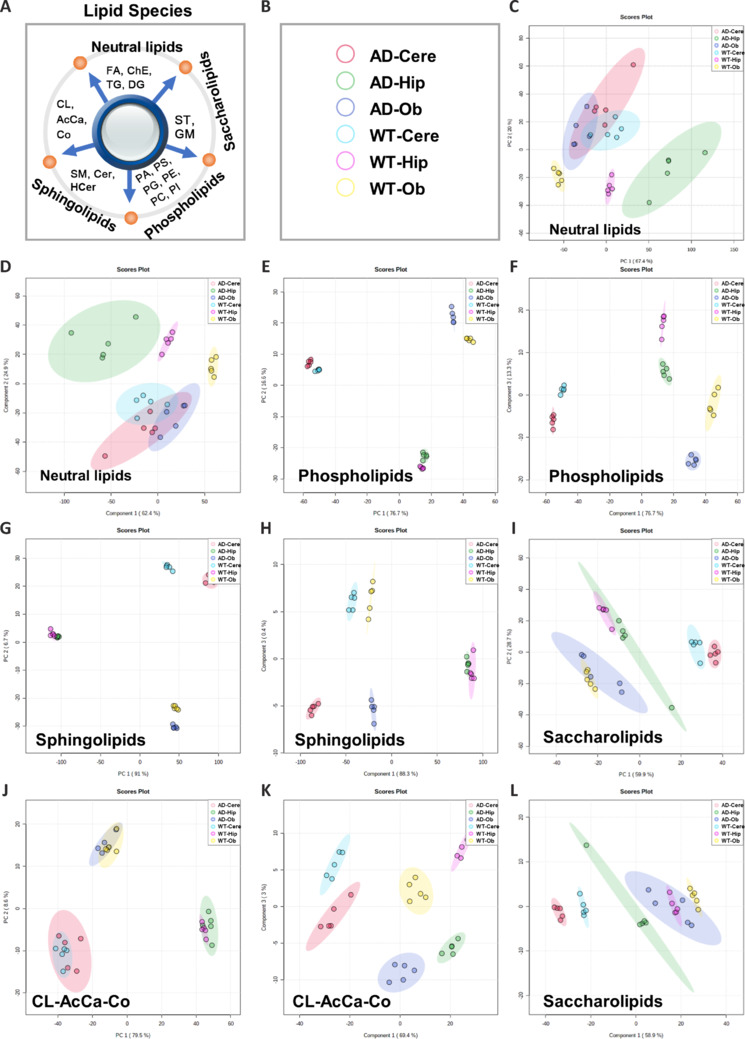
Differential lipid species between AD and WT brain regions. (A) Lipids were classified as five classes, as follows: neutral lipids (FA, ChE, TG, DG), saccharolipids (ST), Phospholipids (PA, PS, PG, PE, PC, PI), sphingolipids (SM, Cer, HCer), others (CL, AcCa, Co, mitochondria-associated lipid). (B) The label color corresponding to the group. (C, E, G, I, J) A two-dimensional PCA plot of the different lipid species, neutral lipids (C), phospholipids (E), sphingolipids (G), saccharolipids (I) and others (J). (D, F, H, K, L) OPLS-DA plot of the different lipid species, neutral lipids (D), phospholipids (F), sphingolipids (H), saccharolipids (L) and others (K). AcCa: Acylcarnitine; AD: Alzheimer’s disease; Cer: ceramide; Cere: cerebellum; ChE: fatty acid; CL: cardiolipin; Co: coenzyme; DG: diacylglycerol; FA: fatty acid; HCer: hexosylceramide; Hip: hippocampus; Ob: olfactory bulb; OPLS-DA: orthogonal partial least-squares discriminant analysis; PA: phosphatidic acid; PC: phosphatidylcholine; PCA: principal component analysis; PE: phosphatidylethanolamine; PG: phosphatidylglycerol; PI: phosphatidylinositol; PS: phosphatidylglycerol; SM: sphingomyelin; ST: sterol lipid; TG:triglyceride phosphate; WT: wild type.

As AD progresses, different brain regions show varying degrees of injury (Bancroft et al., 2019). However, research on the possible relationship between lipid changes and injury in these regions has been limited. Therefore, we employed PCA and OPLS-DA to investigate the lipid composition of the three brain regions in WT and AD mice, which revealed well-dispersed clusters (**Additional Figure 3A** and **B**). To comprehensively evaluate the degree of change in specific lipid species, the lipids were classified into five categories: neutral lipids, saccharolipids, phospholipids, sphingolipids, and others (mitochondria-associated lipids) (**Figure 3A** and **B**). The PCA data showed obvious dispersal of phospholipids and sphingolipids among the six groups (**Figure 3C**, **E**, **G**, **I**, and **J**). Classification by OPLS-DA further revealed evidence of differences in saccharolipids and mitochondria-associated lipids among the six groups (**Figure 3D**, **F**, **H**, **K** and **L**). However, no clear between-group differences in neutral lipids were detected in the PCA or OPLS-DA (**Figure 3C** and **D**).

**Figure 4:**
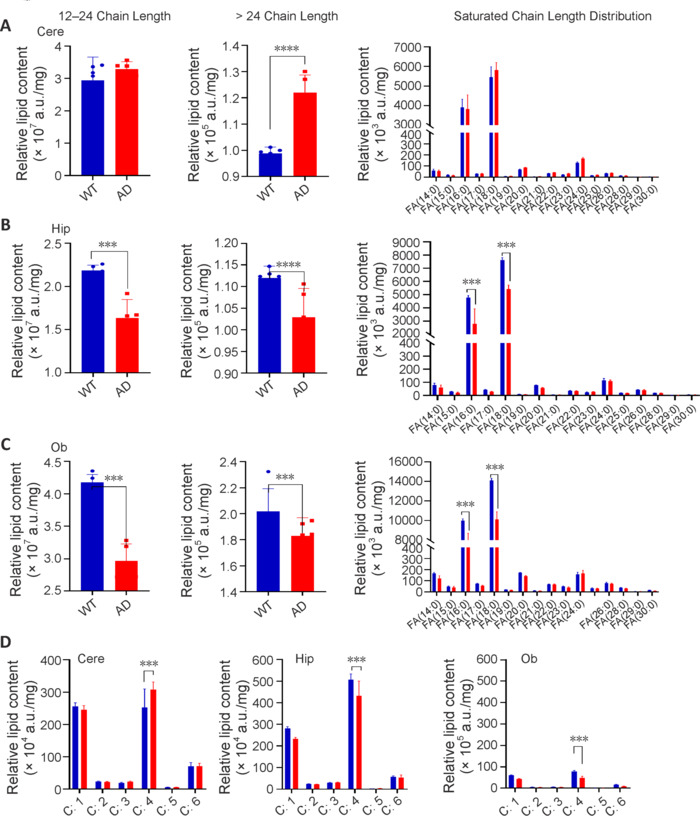
Fatty acyl chain length variation in distinct brain regions. (A–D) The mean peak intensities of FA—medium- and long-chain FA (C12 to C24), long-chain FA (> C24), and saturated chain length distribution — between WT and AD. (A) Cere, (B) Hip, (C) Ob, (D) number of double bonds. Data are presented as mean ± SEM. Unpaired Student’s *t*-test, ***P < 0.001, ****P < 0.0001. AD: Alzheimer’s disease; Cere: cerebellum; FA: fatty acid; Hip: hippocampus; Ob: olfactory bulb; WT:wild type.

Next, we further subdivided the neutral lipids into FAs and ChE-DG-TG. On the basis of the PCA profile, there were clear differences in FAs between the WT and AD groups in the Hip and Ob, but not in the Cere (**Additional Figure 3A–D**). Therefore, we further analyzed the FAs by chain length. Categorizing the FAs into medium-to-long-chain FAs (C12 to C24) and long-chain FAs (> C24), we observed a statistically insignificant greater peak intensity for long-chain FAs in the AD Cere compared with that in the WT Cere (**Figure 4A**). By contrast, the concentrations of both medium-to-long-chain FAs and long-chain FAs in the Hip and Ob regions were lower in AD mice than in WT mice (**Figure 4B** and **C**). Further examination of the FA chain distribution revealed that the chain lengths mainly comprised 16:0 and 18:0, and that the number of double bonds was enriched in C:1 and C:4. Compared with WT mice, AD mice exhibited higher proportions of unsaturated FAs in the Cere and Hip, but a lower proportion of unsaturated FAs in the Ob (**Figure 4D**).

Using a false discovery rate-corrected *P* value cutoff of ≤ 0.05 and a FC of ≥ 1.3 or ≤ 0.7, we identified five molecules with between-group alterations in all three regions: acylcarnitine (AcCa; 18:1), monosialotetrahexosylganglioside (GM3; d36:1), HexCer (d18:1/23:2), SM (d18:1/21:5), and sphingosine (d18:1) (**Additional Figure 2**). Five molecules were altered both in Cere and Hip: AcCa (16:0), ceramide (d18:0/18:0), ceramide (d18:1/22:1), DG (18:1/24:0), and DG (18:1/24:1). Three molecules were altered both in Cere and Ob: ChE (20:4), TG (16:0e/20:1/20:1), and TG (18:0e/20:1/20:1). Five molecules were altered both in Hip and Ob: ChE (22:4), ChE (22:6), HexCer (d18:1/22:2), SM (d35:1), and sulfatide (d18:1/18:0). Taken together, these results demonstrated significantly different lipid compositions between the AD and WT groups, with the separation between the Hip and Ob regions being even more evident than that of the Cere region.

### Potential lipid pathway of Alzheimer’s disease progression

**Figure 5:**
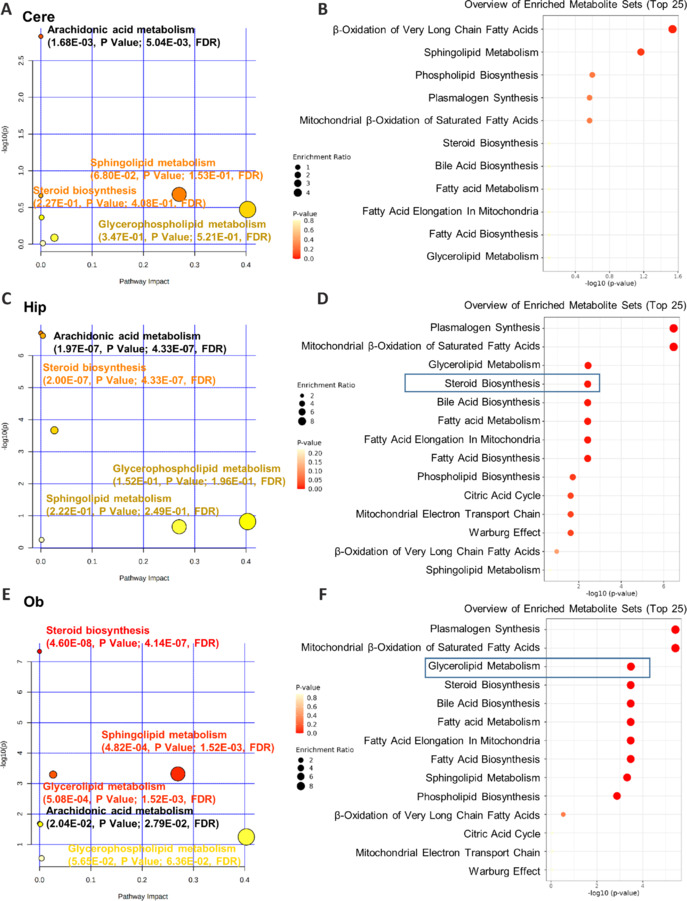
Global pathway analysis of lipidomics in distinct brain regions. (A–F) A bubble chart representation of KEGG pathways and enriched pathways of the different brain regions, Cere (A, B), Hip (C, D), and Ob (E, F) generated with MetaboAnalyst 5.0 using the peak intensity data. Cere: Cerebellum; Hip: hippocampus; KEGG: Kyoto Encyclopedia of Genes and Genomes; Ob: olfactory bulb.

Metabolic pathway analysis and multiple comparison testing of the biomarker candidates demonstrated significant changes (*P* < 0.05) in the metabolism of various lipids, including arachidonic acid, glycerophospholipids, sphingolipids, and steroids, in AD mice (**Figure 5A–F**). Specifically, in the AD Cere and Hip regions, biosynthesis of steroids and metabolism of arachidonic acid, sphingolipids, and glycerophospholipids were significantly impacted. In the AD Ob, biosynthesis of steroids and metabolism of sphingolipids, glycerolipids, glycerophospholipids, and arachidonic acid were significantly impacted. These results aligned with the altered metabolic pathways observed in AD mice and patients (Zhang et al., 2020). In addition to changes in the metabolism of sphingolipids and glycerophospholipids, pathway analysis showed significant enrichment in the mitochondrial electron transport chain, citric acid cycle, and steroid biosynthesis (**Figure 5D–F**). Cholesterol plays a vital role in the pathophysiological mechanisms of AD, impacting metabolism in the brain either directly (through oxysterols) or indirectly (through apolipoprotein E [APOE]) to cause neurodegeneration. To our knowledge, steroid biosynthesis has not been assessed using an untargeted method in AD mice. Therefore, we sought to perform a sterol analysis of the AD mouse brain using untargeted MS technology.

### Differences in regional sterol content of the Alzheimer’s disease brain

**Figure 6:**
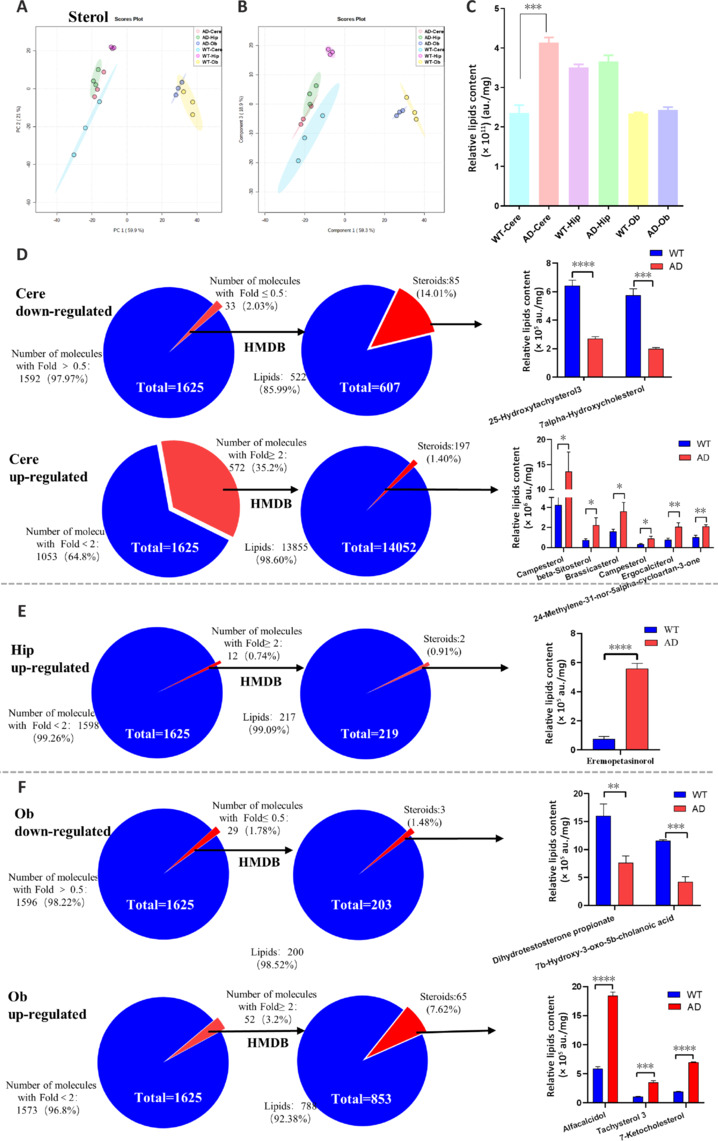
Analysis of sterol in distinct brain regions. (A, B) A two-dimensional PCA (A) and OPLS-DA (B) plot of the sterol in distinct brain regions. (C) Total sterol (overall mean peak intensity of all sterol) between WT and AD. (D) The pie graph is the percentage of eligible sterol in Cere after screening (left), presentation of the level of screened sterol (right). (E) The pie graph is the percentage of eligible sterol in Hip after screening (left), presentation of the level of screened sterol (right). (F) The pie graph is the percentage of eligible sterol in Ob after screening (left), presentation of the level of screened sterol (right). Data are presented as mean ± SEM. Unpaired Student’s *t*-test, *P < 0.05, **P < 0.001, ***P < 0.001, ****P < 0.0001. AD: Alzheimer’s disease; Cere: cerebellum; Hip: hippocampus; Ob: olfactory bulb; OPLS-DA: orthogonal partial least-squares discriminant analysis; PCA: principal component analysis; WT: wild type.

We acquired 1625 different compounds for UHPLC-MS analysis of sterols. The data from PCA and OPLS-DA indicated clear differences in sterols in the Cere and Hip regions between WT and AD mice, demonstrating a scaling relationship (**Figure 6A** and **B**). Although the relative sterol level in the Cere region was higher in AD mice than in WT mice (**Figure 6C**), the structural complexity of sterols makes such an analysis challenging. To overcome this, we screened potential compounds based on their molecular weight (m/z) standards, using the following criteria: FC of < 0.5 or > 2 for the AD/WT groups and a *P* value < 0.05.

For the Cere region, we identified 33 downregulated molecules and 572 upregulated molecules. Among the downregulated and upregulated molecules, 607 and 14,052 potential molecules were identified through HMDB screening, of which 522 and 13,855 were identified as lipids and excluded, respectively, leaving 85 downregulated and 197 upregulated sterols for further analysis (**Figure 6D**). Similar HMDB screening of the 12 upregulated molecules in the Hip region identified 219 potential molecules, of which 217 were excluded and the remaining two were further analyzed (**Figure 6E**). In the Ob region, 29 downregulated and 52 upregulated molecules led to the identification of 203 and 853 potential molecules, of which 200 and 788 were excluded, respectively, leaving three downregulated and 65 upregulated sterols for further analysis (**Figure 6F**).

The pie charts shown in **Figure 6D–F** illustrate the upregulated and downregulated sterols in each brain region that met our selection criteria following alignment of the candidate molecules on the HMDB website. In the Cere region, we identified two downregulated molecules: 7α-hydroxycholesterol (7α-HC; m/z = 385.347605) and 25-hydroxytachysterol (25-HC; m/z = 383.331555). 7α-HC is an oxysterol that has been proposed as a biomarker for lipid peroxidation and has been shown to accumulate within atherosclerotic plaques, contributing to inflammatory signaling in diseased arteries (Kitano et al., 2007). Its association with AD progression makes it a potential target for further investigation (Erridge et al., 2007). Additionally, its isomer, 7β-hydroxycholesterol, was recently identified as a pathologically relevant peripheral biomarker of AD in triple-transgenic AD mice (Ha et al., 2024). We also identified six upregulated molecules in the Cere region: brassicasterol (m/z = 381.352525), campesterol (m/z = 383.368005), isofucosterol/stigmasterol (m/z = 395.368295), ergocalciferol (m/z = 397.347225), beta-sitosterol (m/z = 397.383875), and 24-methylene-31-nor-5alpha-cycloartan-3-one (24m-5α-cyclo) (m/z = 407.368835). Brassicasterol, an ergosterol derivative, has been identified as a potential cerebrospinal fluid biomarker for AD (Vanmierlo et al., 2011). Campesterol, a phytosterol, helps to control total cholesterol levels and modifies the levels of high-density lipoprotein, low-density lipoprotein, and triacylglycerol (Juvik et al., 2017). Beta-sitosterol, a main dietary phytosterol found in plants, has anti-carcinogenic and anti-atherogenic properties (Maguire et al., 2003). Ergocalciferol is an active form of vitamin D and may play a role in brain function. We were unable to differentiate between isofucosterol and stigmasterol because of their identical molecular weights.

In the Hip region, we identified one upregulated molecule that has not been previously associated with any disease: eremopetasinorol (m/z = 226.1811). In the Ob region, we identified two downregulated molecules: dihydrotestosterone propionate (m/z = 329.2484) and 7β-hydroxy-3-oxo-5b-cholanoic acid (m/z = 385.2748). Dihydrotestosterone propionate affects dominance and sexual behaviors, and is not a naturally occurring metabolite (Cochran and Perachio, 1977). 7β-Hydroxy-3-oxo-5b-cholanoic acid is a bile acid. Finally, in the Ob region, we identified three upregulated molecules: alfacalcidol (m/z = 383.3316), tachysterol 3/lumisterol 3 (m/z = 385.3476), and 7-ketocholesterol (m/z = 401.3426). Alfacalcidol is an active metabolite of vitamin D, which reportedly performs essential functions in the brain (Ringe and Schacht, 2007), regulating various brain processes, including neurotransmitter synthesis and metabolism, neurotrophin expression, and neuroinflammation. Alfacalcidol is also involved in calcium homeostasis and bone metabolism. It was challenging to make distinctions between tachysterol 3 and lumisterol 3, but both have been demonstrated to inhibit keratinocyte proliferation (Bocheva et al., 2021). 7-Ketocholesterol is a primary oxidation product of cholesterol (oxysterol) found in human atherosclerotic plaques and was found to be more atherogenic than cholesterol in some animal studies (Brooks et al., 1966; Wang et al., 2017). Additionally, 7-ketocholesterol has been implicated in the pathogenesis of neurodegenerative diseases such as AD, inducing neuronal cell death and promoting the accumulation of Aβ (Mathieu et al., 2008; Ghosh and Khare, 2017).

### Expression levels of genes related to lipid and sterol metabolism

**Figure 7:**
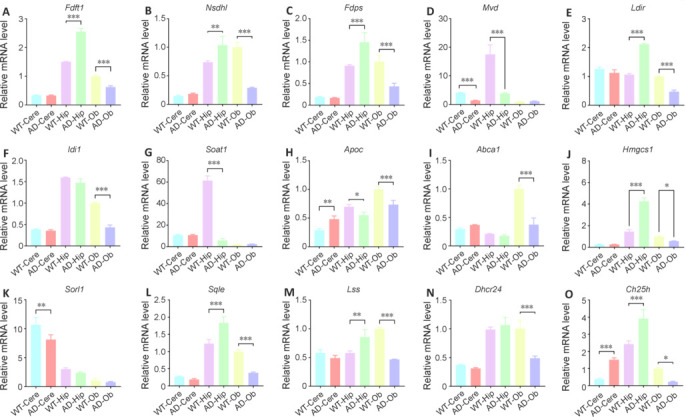
Validation the gene associated lipid and sterol metabolism with by qPCR. (A–O) Relative mRNA expression level of *Fdft1* (A), *Nsdhl* (B), *Fdps* (C), *Mvd* (D), *Ldlr* (E), *Idi1* (F), *Soat1* (G), *Apoc* (H), *Abca1* (I), *Hmgcs1* (J), *Sorl1* (K), *Sqle* (L), *Lss* (M), *Dhcr24* (N), and *Ch25h* (O) in different brain regions (Cere, Hip, and Ob) of WT and AD mice. Data are presented as mean ± SEM. Unpaired Student’s *t*-test, *P < 0.05, **P < 0.001, ***P < 0.001. AD: Alzheimer’s disease; Cere: cerebellum; Hip: hippocampus; Ob: olfactory bulb; WT: wild type.

To investigate whether the observed differences in lipids and sterols were associated with changes in gene expression, we focused on 15 genes known to be involved in the biosynthesis, transport, and regulation of lipid and sterol metabolism in the context of AD (Xu et al., 2008; Malik et al., 2012; Hai and Smith, 2021), validating their expression levels through qPCR. The selected genes, citing the key studies showing their involvement in AD, were as follows: *Fdft1* (Valdez et al., 2011), *Fdps* (Carter, 2008), *Idi1* (Malik et al., 2012), *Ldlr* (Malik et al., 2012), *Mvd* (Bharadwaj et al., 2022), *Mvk* (Malik et al., 2012), *Nsdhl* (Malik et al., 2012), *Apoc* (Petit-Turcotte et al., 2001), *Abca1* (Holstege et al., 2022), *Lss* (Martinez et al., 2023), *Sqle* (Ge et al., 2021), *Dhcr24* (Zhang et al., 2023), *Ch25h* (Shibata et al., 2006), *Sorl1* (Lee et al., 2023), and *Hmgcs1* (Lee et al., 2024). Strikingly, all of these genes were found to be significantly downregulated in the Ob region of AD mice compared with that in WT mice (**Figure 7**), which may have contributed to the abnormal cholesterol metabolism and subsequent lipid accumulation in this brain region.

## Discussion

AD is a neurodegenerative disease that affects memory and other mental functions, and is the leading cause of dementia (Tahami Monfared et al., 2022; Li et al., 2025). In this study, we used the MTBE method to acquire lipids and the classical Bligh and Dyer method to acquire sterols for region-resolved relative quantitative lipid and sterol analysis of the mouse brain.

We specifically chose 8-month-old 5×FAD mice as a representative model because this age corresponds to an AD stage with substantial Aβ deposition, as well as established neuroinflammation and neurodegenerative pathology (Oakley et al., 2006; Choi et al., 2018). This stage in mice is comparable to the middle-to-late stages of AD in humans, allowing us to investigate lipid metabolism changes that are closely associated with AD pathology. Through a comprehensive analysis of lipid content and composition, including sterols, we discerned notable differences in sterols and other lipids between AD-afflicted and WT mice.

Our quantification of 664 lipid species encompassing 23 lipid classes, along with characterization of FA chain length and saturation, revealed that different AD-affected brain regions had distinct feature lipid classes. The PCA results showed marked differences in the lipid composition of these regions, as well as a slightly higher total lipid content in the Cere compared with that in the Hip and Ob. Phospholipids, sphingolipids, saccharolipids, and mitochondria-associated lipids all showed obvious differences between WT and AD mice, with specific elevations in ceramide and SM in AD model mice, consistent with previous findings (Loft et al., 2022). Both ceramide and SM are involved in the metabolism of Aβ, the protein that forms plaques in AD brains, and ceramides may promote its aggregation (Jazvinšćak Jembrek et al., 2015). Ceramide accumulation has also been linked to increased β-secretase activity, leading to elevated production of Aβ peptides. Ceramides are involved in neuroinflammation and neuronal apoptosis, which is a key feature of AD (Lee et al., 2004). Elevated ceramide levels can activate proinflammatory signaling pathways, contributing to the neurodegenerative process (Cutler et al., 2004). Specifically, ceramide is thought to activate NF-κB, leading to the production of inflammatory cytokines and reactive oxygen species, which accelerate neuronal damage. As the precursor of complex sphingolipids, ceramide has been linked with certain psychiatric and neurological disorders, leading researchers to propose that specific ceramide species (e.g., C16:0 and C24:1) have potential as biomarkers of dementia (Brodowicz et al., 2018). The levels of ceramides C18:1, C16:0, and C24:0 are elevated in human plasma, consistent with data for the Cere and Hip regions in our AD model. Similarly, SMs are key components of lipid rafts, which form specialized microdomains in cell membranes. These rafts are important for cell signaling, and their disruption can impact synaptic function and promote the accumulation of Aβ. Elevated SM levels alter the composition and function of these lipid rafts, affecting neuronal signaling and membrane integrity, which may facilitate the formation of Aβ plaques (Crivelli et al., 2020). SM (d18:1/18:0) is significantly increased in the cerebrospinal fluid of AD patients (Koal et al., 2015). Targeting key enzymes in ceramide/SM metabolism, such as sphingomyelinases and ceramide synthases, offers the potential to mitigate neuronal damage and modulate amyloidogenic processes, providing new avenues for AD intervention (Baloni et al., 2022).

GM3 is elevated in the brains of AD patients and AD model mice, with serum levels positively associated with disease severity in patients (Dodge et al., 2022). In addition, we found that sulfatide levels in the Hip and Ob regions were lower in AD mice than in WT mice, consistent with a report of sulfatide loss in an early AD model (Han, 2010). Additionally, Proitsi et al. (2015) reported that long-chain ChEs were reduced in the serum of AD patients, consistent with our results in AD mice. We found that AD model mice had elevated brain levels of TG and DG, which have been closely associated with dyslipidemia and shown to be elevated in the serum in some neurological disorders, such as frontotemporal dementia (Kim et al., 2018) and AD in mice (Burgess et al., 2006). Another study indicated that medium-chain triglycerides improved cognitive functioning in patients with mild-to-moderate AD (Henderson et al., 2009). However, a study by Sharma et al. (2014) concluded that, in the absence of routine genomic profiling, medium-chain triglycerides play a minimal role in clinical practice.

Recent studies have shown that the brain uses approximately 20% of the body’s daily energy, which is produced in the form of ATP in the mitochondria. Our PCA and OPLS-DA of lipid clusters in WT and AD mouse brains confirmed the dispersal of mitochondria-associated lipids, such as cardiolipin and coenzyme A, in both groups. Energy is derived from FA oxidation, and essential polyunsaturated FAs are critical for brain development and maintenance. Abnormalities in polyunsaturated FAs have been associated with neuropsychiatric disorders, such as depression, AD, and bipolar disorder (Milte et al., 2009). We also evaluated the FA chains and levels of unsaturated FAs. The lengths of medium and long chains were lower in AD mice than in WT mice in both the Hip and Ob regions, indicating a different energy level in the AD-afflicted brain. The lipid content and BioPAN analysis further showed that the metabolism of phospholipids and sphingolipids were significantly altered in AD mice. Pathway analysis revealed significant enrichment of arachidonic acid metabolism, sphingolipid metabolism, and glycerophospholipid metabolism in AD mice compared with that in WT mice, consistent with a previous report (Zhang et al., 2020). Additionally, steroid biosynthesis was found to be significantly altered in AD mice, both in the pathway analysis and in the enriched metabolite pathway.

To delineate the role of sterols in AD progression, we specifically extracted and analyzed sterols in brain tissues, revealing significant differences between AD and WT mice. By eliminating other lipid molecules and aligning each candidate on the HMDB website, we identified potential AD-related sterol molecules and supplied the corresponding fragmentation (MS/MS) data (**Additional Figure 4**). In the Cere region, we observed downregulation of 7α-HC and 25-hydroxytachysterol, both of which are associated with inflammatory signaling (Kitano et al., 2007; Wong et al., 2020). Notably, 7α-HC has potential relevance as a biomarker for AD progression (Ha et al., 2024). Several sterols that were upregulated, including brassicasterol, campesterol, and β-sitosterol, have effects on lipid metabolism and inflammation (Maguire et al., 2003; Vanmierlo et al., 2011) that suggest possible roles in AD pathology. In the Hip region, we identified upregulation of eremopetasinorol, though its disease relevance remains unclear. The Ob region exhibited both downregulated (dihydrotestosterone propionate and 7β-hydroxy-3-oxo-5β-cholanoic acid) and upregulated (alfacalcidol, tachysterol/lumisterol, and 7-ketocholesterol) sterols, with 7-ketocholesterol being a notable contributor to neuroinflammation and Aβ accumulation in AD. To the best of our knowledge, this is the first study to use untargeted lipidomic analysis to demonstrate that abnormal sterol metabolism plays a role in AD progression. Meanwhile, we observed differential expression of 15 genes involved in lipid and sterol metabolism (*Fdft1*, *Fdps*, *Idi1*, *Ldlr*, *Mvd*, *Mvk*, *Nsdhl*, *Apoc*, *Abca1*, *Lss*, *Sqle*, *Dhcr24*, *Ch25h*, *Sorl1*, and *Hmgcs1*). We found that all of these genes were significantly downregulated in the Ob of AD mice, which may have contributed to the abnormal cholesterol metabolism and subsequent lipid accumulation in this brain region. Taken together, our findings have validated the hypothesis that sterols and other lipids change during the disease progression of AD.

Despite these findings, our study also had several limitations. First, given the limited availability of AD model mice, the investigation relied on a relatively small number of samples collected at a single time point. Second, while our lipidomic profiling was comprehensive for other lipid classes, the analysis of sterols was limited to untargeted approaches. Untargeted sterol analysis, although powerful for discovering novel sterol species and gaining a broad overview of sterol metabolism, lacks the sensitivity and specificity required for accurate quantification. This is particularly relevant in complex brain tissue samples with regional variations in sterol concentrations. Furthermore, sterol species often overlap with other lipid classes, complicating their identification on the basis of m/z values and retention times alone. To strengthen our conclusions and enhance the understanding of the role of sterols in AD, future studies aimed at providing precise quantification of individual sterol species should include targeted methods, such as gas chromatography-MS or LS-MS/MS. These methods have shown promise in neurodegenerative diseases such as AD, where the key sterols 7β-hydroxycholesterol and 24S-hydroxycholesterol have been validated as biomarkers (Schönknecht et al., 2002; Ha et al., 2024). Third, to gain a more nuanced understanding of how each of the differentially expressed genes identified here influence lipid metabolism and the metabolic alterations and functional outcomes in AD, targeted analyses and transcriptional regulation studies are crucial. Despite these limitations, we have provided detailed lipidomic and sterolomic profiles of 5×FAD model mice, significantly contributing to the knowledge gap surrounding lipid and sterol metabolism in AD.

The following points highlight the innovation of our study. First, untargeted lipidomics and sterolomics were performed on three different brain regions (Cere, Hip, Ob), and untargeted sterolomics of AD has not been reported in previous studies. We discovered that the metabolism of lipids, including specific sterol species, was abnormal in all three brain regions of AD mice. Second, we found that the total lipid content in the Cere region was significantly higher in AD mice than in WT mice, while the level of sterols in the Ob region was significantly higher in AD compared with WT. Furthermore, we found that the levels of medium- and long-chain FAs were higher in the Hip and Ob regions of AD mice, and the levels of unsaturated FAs were lower in AD, compared with WT. Additionally, we observed significant differences in the levels and metabolism of phospholipids and sphingolipids between AD and WT mice. This finding may lead to the development of potential biomarkers for AD. Third, qPCR analysis of the expression of genes related to lipid and sterol metabolism revealed abnormalities in different regions of the AD mouse brain. In conclusion, our results provide novel insights into AD progression, indicating that abnormalities in lipid and cholesterol metabolism may collectively account for the pathophysiological mechanisms of AD. Further studies are needed to elucidate the underlying mechanisms and identify new therapeutic targets for treatment and prevention of AD.

## Additional files:

***Additional Figure 1:*** *General overview of experimental flowchart.*

Additional Figure 1 General overview of experimental flowchart.
Cere: Cerebellum; Hip: hippocampus; Ob: olfactory bulb; RT-PCR: quantitative polymerase chain reaction; WT: wild type.

***Additional Figure 2:*** *General overview of lipids between AD and WT brain regions.*

Additional Figure 2 General overview of lipids between AD and WT brain regions.
(A, D, G) A two-dimensional PCA plot of the different brain regions, Cere (A), Hip (D), and Ob (G), was generated with MetaboAnalyst 5.0 using the peak intensity data. (B, E, H) OPLS-DA plot of the different brain regions, Cere (B), Hip (E), Ob (H). (C, F, I) Scatterplot depicting lipid abundance (peak intensity) in different brain regions, Cere (C), Hip (F), Ob (I) of AD versus WT. Red dots indicate lipids that are significantly enriched (fold change > 2 and *P* < 0.01) in AD compared to WT. Blue dots indicate lipids that are significantly depleted (fold change < 0.5 and *P* > 0.01) in AD compared to WT. Light gray dots indicate lipids that are either not significantly enriched or fail to meet the fold change requirement. AD: Alzheimer's disease; Cere: cerebellum; FC: fold change; FDR: false discovery rate; Hip: hippocampus; Ob: olfactory bulb; OPLS-DA: orthogonal partial least-squares discriminant analysis; PCA: principal component analysis; WT: wild type.

***Additional Figure 3:*** *PCA and OPLS-DA plots of all lipids and FA in distinct brain regions.*

Additional Figure 3 PCA and OPLS-DA plots of all lipids and FA in distinct brain regions.
(A, B) PCA (A) and OPLS-DA (B) plots of all lipids in distinct brain regions. (C, D) PCA (C) and OPLS-DA (D) plots of FA in distinct brain regions. AD: Alzheimer's disease; Cere: cerebellum; Hip: hippocampus; Ob: olfactory bulb; OPLS-DA: orthogonal partial least-squares discriminant analysis; PCA: principal component analysis; WT: wild type.

***Additional Figure 4:*** *Mass spectrometry peak profiles of brain region-specific sterols in Alzheimer’s disease mice.*

Additional Figure 4 Mass spectrometry peak profiles of brain region-specific sterols in Alzheimer's disease mice.

***Additional Table 1:*** *The primer sequences for quantitative polymerase chain reaction.*

***Additional Table 2:*** *Lipid species percentage in distinct brain regions.*

## Data Availability

*All data relevant to the study are included in the article or uploaded as Additional files*.
