# Supplementary figures and images for: Lipidomic and sterolomic profiles of different brain regions in the mouse model of Alzheimer’s disease
Source: Neural Regen Res. 2025 Feb 24;21(6):2543–52. doi: 10.4103/NRR.NRR-D-24-00975 (PMC13211812; doi:10.4103/NRR.NRR-D-24-00975)

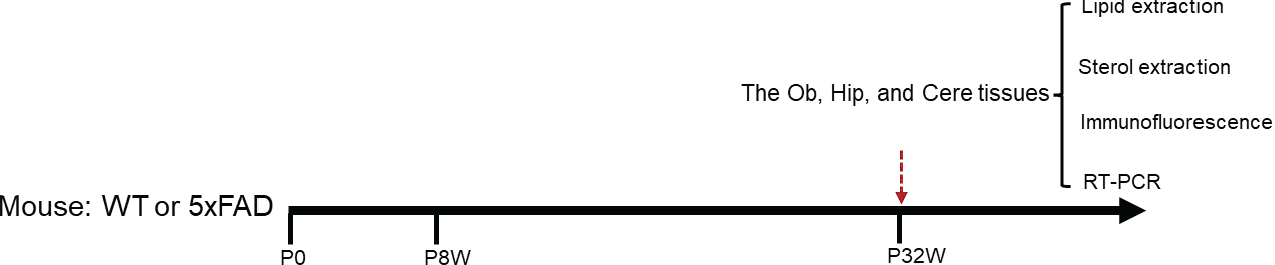

Supplement: Supplementary file 1 [file NRR-21-2543_Suppl1.tif]

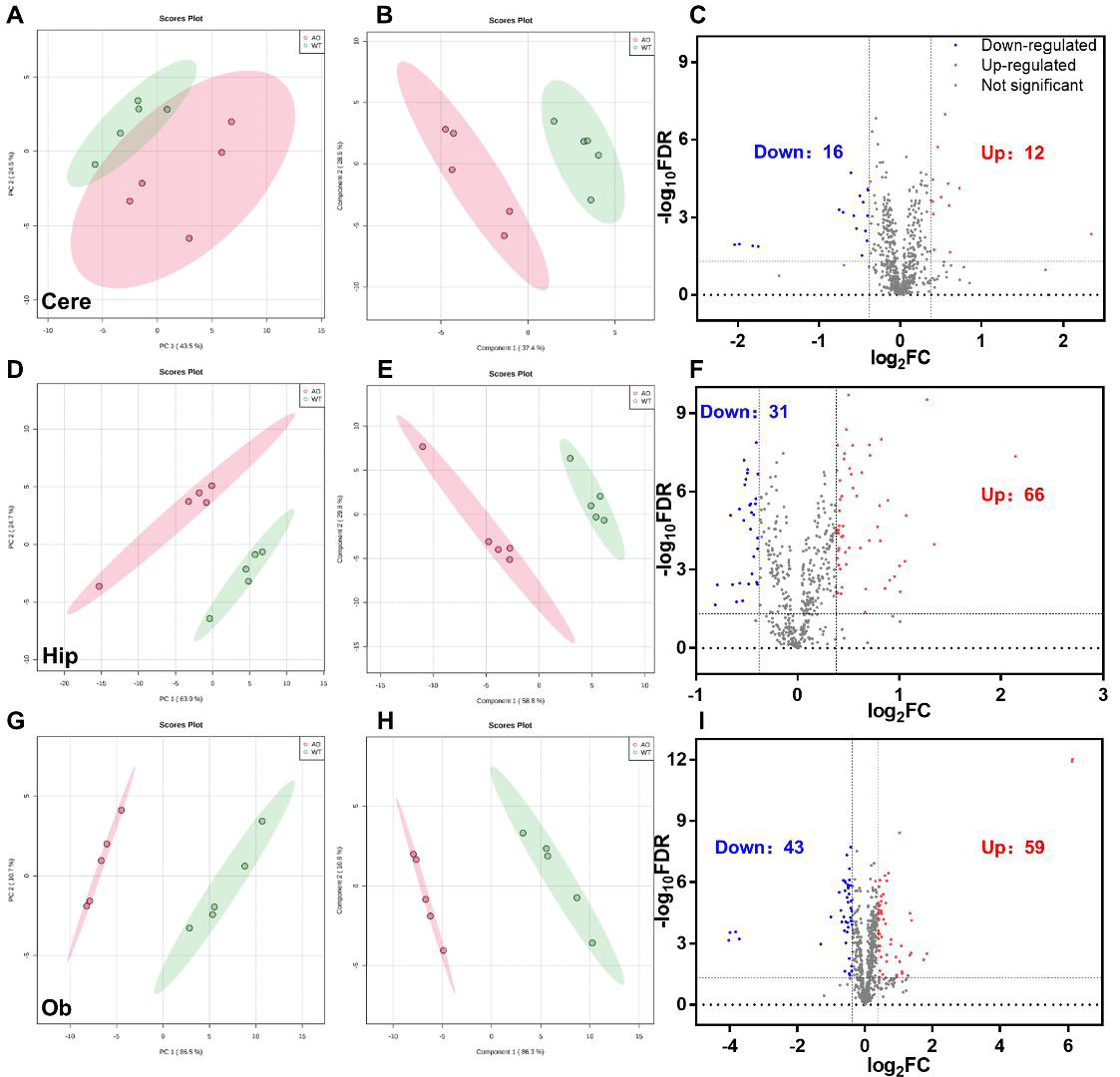

Supplement: Supplementary file 2 [file NRR-21-2543_Suppl2.tif]

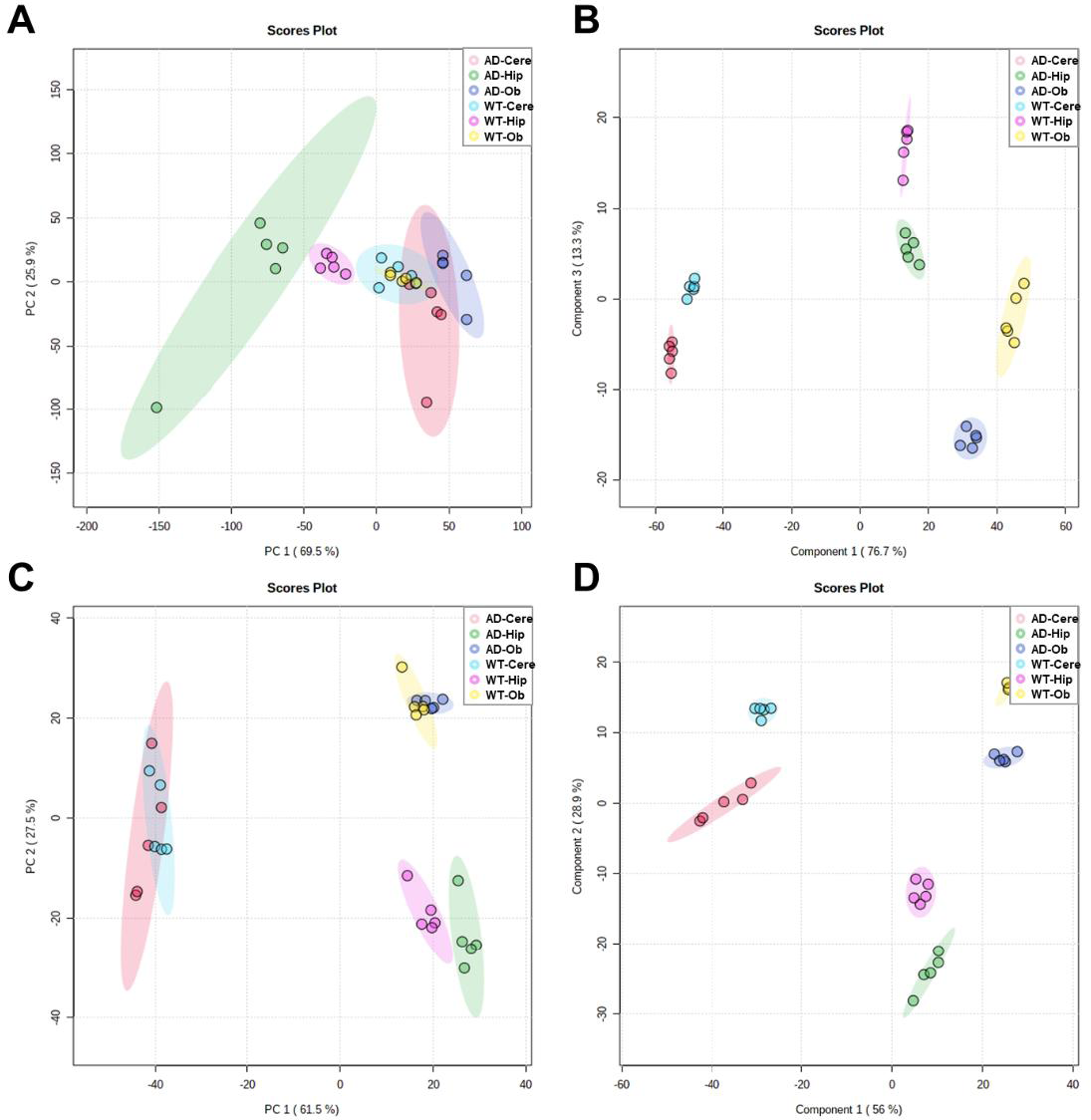

Supplement: Supplementary file 3 [file NRR-21-2543_Suppl3.tif]

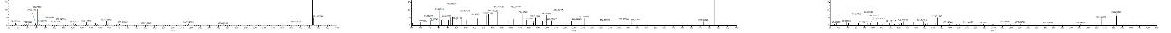

Supplement: Supplementary file 4 [file NRR-21-2543_Suppl4.tif]
